# A new freshwater snail genus of Squamapicidae (Gastropoda, Truncatelloidea) with a novel posterior respiratory canal unveils an unexpected morphological innovation

**DOI:** 10.3897/zookeys.1282.186276

**Published:** 2026-06-10

**Authors:** Hui Zheng, Yu-Hui Xing, Le-Jia Zhang, Qian Li, Zhong-Guang Chen, Hong-Quan Xiang, Shan Ouyang, Xiao-Chen Huang, Xiao-Ping Wu

**Affiliations:** 1 School of Life Sciences, Nanchang University, Nanchang, 330031, China School Life Sciences, Nanjing Normal University Nanjing China; 2 School Life Sciences, Nanjing Normal University, Nanjing, 210023, China School of Zhongbei, Nanjing Normal University Zhenjiang China; 3 School of Zhongbei, Nanjing Normal University, Zhenjiang, 212300, China Kunming Institute of Zoology, Chinese Academy of Sciences Kunming China; 4 Kunming Institute of Zoology, Chinese Academy of Sciences, Kunming 650023, China School of Life Sciences, Nanchang University Nanchang China; 5 Department of Animal Science, Yuxi Agriculture Vocation-Technical College, Yuxi, 653106, China Department of Animal Science, Yuxi Agriculture Vocation-Technical College Yuxi China

**Keywords:** Convergent evolution, freshwater snails, microgastropods, new genus, new species, shell morphology, systematics, Truncatelloidea, Yunnan-Guizhou Plateau

## Abstract

Highly ornamented protoconchs and a developed posterior canal are exceptionally rare and unique shell characteristics in strict freshwater gastropods. This study combines morphological and molecular phylogenetic approaches to describe a new genus and species within the Squamapicidae family that possesses the characteristic of a highly ornamented protoconch: *Canglangia heyuemingi* H. Zheng, H.-Q. Xiang, L.-J. Zhang & X.-P. Wu, **gen. et sp. nov**. Through the observation and cultivation of three species of freshwater snails with highly ornamented protoconchs from Yunnan, it was found that the formation and function of the highly ornamented protoconch vary across different species. The role of the posterior canal is discussed by examining the habitat of *C. heyuemingi***gen. et sp. nov**.

## Introduction

Freshwater snails constitute a vital yet underappreciated component of global aquatic biodiversity, playing critical roles in nutrient cycling, sediment stability, and food webs as primary consumers and prey species (Dillon 2000; Du et al. 2019; Zheng et al. 2026). However, research efforts have historically prioritized larger, charismatic fauna, leaving significant gaps in our understanding of microgastropods (Strong et al. 2008; Zhang et al. 2024; Czaja et al. 2025). These small-bodied organisms, though easily overlooked due to their size and often cryptic habitats, exhibit remarkable phylogenetic diversity, high degrees of endemism, and peculiar morphological adaptations (He et al. 2025; Chen et al. 2025). These snails are sensitive bioindicators and represent unique evolutionary lineages (Strong et al. 2008). Yet, they face severe threats from habitat degradation, pollution, and climate change (Du et al. 2019; Neubauer et al. 2021), with many becoming extinct before formal study (He et al. 2025; Xiang et al. 2026). Research into these diminutive organisms is thus an imperative for biodiversity conservation, not merely a taxonomic exercise.

A prime example of such overlooked diversity is found within the superfamily Truncatelloidea, a large and successful radiation of microsnails that has extensively colonized freshwater, marine, and even damp terrestrial environments worldwide, giving them a high level of diversity in shell morphology (Cummings and Lydeard 2019). Despite this, highly ornamented protoconchs are extremely rare in strictly freshwater Truncatelloidea and even among all strictly freshwater gastropods (Zhang et al. 2024). This shell structure typically appears in marine or migratory gastropods with veliger larvae (Nützel 2014). Within this superfamily, the family Squamapicidae stands out as one of the most enigmatic and relictual lineages. It has been considered a monotypic group, with its sole known species, *Squamapex taiji* Zhang & von Rintelen, 2024, inhabiting the ancient Lake Fuxian (Zhang et al. 2024).

This study reports the first recorded instance of a strictly freshwater snail possessing both a developed posterior canal, a feature typically associated with marine gastropods and terrestrial pulmonates (Latiolais et al. 2006; Jirapatrasilp et al. 2022, 2025)—and a highly ornamented protoconch. The specimen was collected from montane streams in Luquan County, Yunnan, China. Based on morphological and molecular analyses, we identify it as a new genus and species within the relict family Squamapicidae. We herein provide the formal description of this novel taxon and discuss the potential function of its posterior canal.

## Materials and methods

### Specimen sampling and morphological observations

**Figure 1.**
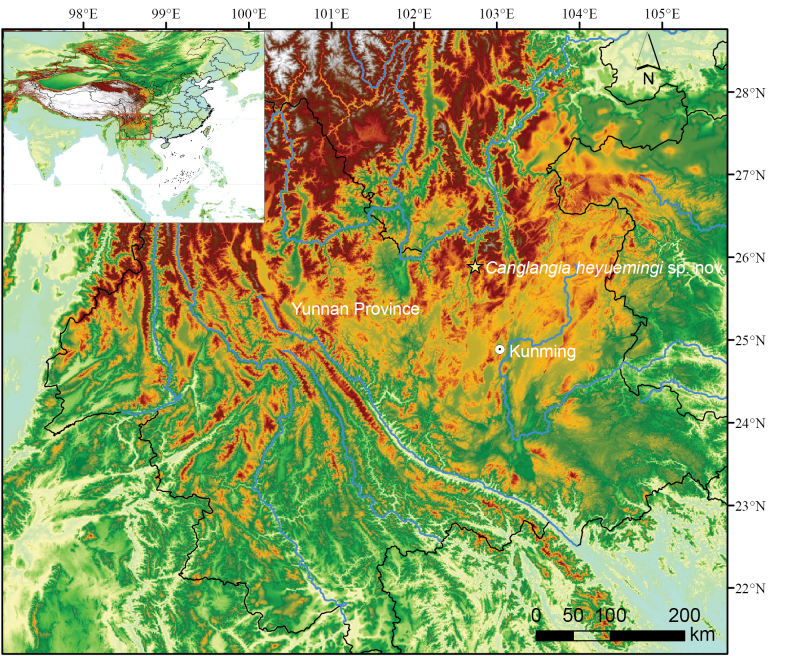
Collection sites of *Canglangia heyuemingi* gen. et sp. nov. The gold star indicates the type locality.

Specimens were collected from a spring near the Pudu River, Luquan Yi and Miao Autonomous County, Kunming City, Yunnan Province, China, in April, July and August 2025 (Fig. 1). The specimens were preserved in 95% ethanol, and deposited at the Museum of Biology, Nanchang University (NCUMB), China. A Vernier caliper was used to measure shell height (H), defined as the maximum dimension parallel to the coiling axis, and shell width (W), defined as the maximum dimension perpendicular to H, following the measurement scheme of Chen et al. (2023). Shells were cleaned using a fine brush, flushed with distilled water, and photographed under a stereomicroscope (Nikon SMZ645). Radulae were dissected from the buccal mass and cleaned enzymatically with proteinase K, sonicated, then mounted on aluminium specimen stubs with adhesive pads. Shells, opercula and radulae were observed by scanning electron microscopy (ZEISS G300).

### Phylogenetic analyses

Total genomic DNA was extracted from foot tissues using a TIANamp Marine Animals DNA Kit (Tiangen Biotech) following the manufacturer’s protocol. Partial sequences of COI, 16S, H3, and 28S were amplified using the following primers: LCO1490 and HCO2198 for COI (Folmer et al. 1994), 16Sar and 16Sbr for 16S (Kessing et al. 1989), H3F and H3R for H3 (Colgan et al. 2000), and 28SD1and 28ff for 28S (Hillis and Dixon 1991; Colgan et al. 2003). Each PCR was performed in a total volume of 20 μL, including 9 μL of PCR mix, 8 μL of double-distilled water, 1 μL of each primer, and 1 μL of the template DNA. The PCR conditions were as follows: initial denaturation at 95 °C for 3 min; 35 cycles of denaturation at 95 °C for 40 s, annealing at 55 °C for 16S (52 °C for COI; 59 °C for 28S; 62 °C for H3) for 30 s, and extension at 72 °C for 30 s for COI and 16S (60 s for H3 and 28S); and final extension at 72 °C for 7 min. Both ends of the sequences were obtained by automated sequencing using Applied Biosystems 3730 in Sangon Biotech Co. Ltd (Shanghai, China). Sequences were aligned using MAFFT v. 7.505 based on the L-INS-i method (Katoh and Toh 2008). In addition, COI and H3 were codon aligned by MUSCLE (Edgar 2004) implemented in MEGA X (Kumar et al. 2018). Gblocks v. 0.91b (Castresana 2000) was used to exclude ambiguous regions from the 16S and 28S alignments. Sequences were concatenated in Phylosuite v. 2.3 (Zhang et al. 2020). All samples and GenBank accession numbers are provided in Suppl. material 1. The best partition schemes and best-fitting substitution models were selected using a greedy search with unlinked branch lengths in PartitionFinder 2 (Lanfear et al. 2017). Bayesian inference (BI) and maximum-likelihood (ML) analysis were performed using MrBayes (v. 3.2.6; Ronquist et al. 2012) and IQ-TREE (v. 2.2; Minh et al. 2020), respectively, with the following partition scheme and models: COI codon1, COI codon2, H3, and 28S under the GTR+F+I+G4 model; COI codon3 under the HKY+F+G4 model; and 16S under the GTR+F+I+G4 model. For Bayesian analysis, two runs were performed simultaneously with four Markov chains starting from a random tree. Bayesian posterior probabilities (BPPs) of nodes were determined using Metropolis-coupled Markov chains (one cold chain) for 10 million generations, with sampling every 1000 generations. The first 25% of sampled trees were discarded as burn-in when the standard deviation of split frequencies of the two runs was less than 0.01; the remaining trees were then used to create a 50% majority-rule consensus tree and to estimate BPPs. Node support for maximum-likelihood analysis was determined using 1000 rapid bootstrap (BS) replicates.

### Divergence time estimation

Divergence times were estimated using BEAST based on our multilocus dataset, incorporating an uncorrelated log-normal relaxed clock model and a calibrated Yule model. Substitution models assigned to each partition are consistent across the phylogenetic analysis. Two priors were used to calibrate the divergence times on the tree based on fossil records. The root of the superfamily Truncatelloidea was calibrated using its oldest and most taxonomically reliable fossil, †*Triasamnicola pilsbryi* Yen & Reeside, 1946, from the Late Triassic Chinle Formation (Utah, United States). To incorporate the minimum age constraint from this fossil, a log-normal distribution was applied as the node-age prior. The parameters were set with an offset of 201.0 Ma, representing the hard minimum bound. The mean (M) was set to 2.0, and the standard deviation (S) to 0.5, with the mean set in real space. The most recent common ancestor (MRCA) of the subfamily Hydrobiinae was calibrated based on the earliest known fossil record of the subfamily, *Polycirsus varicosus*, from the Eocene (Lutetian stage, 41–48 Ma). A log-normal prior was set for this node with an offset of 41.2 Ma, a mean (M) of 2.0, and a standard deviation (S) of 1.0, with the mean set in real space. Four independent MCMC runs were executed for 200 million generations each, sampling every 20, 000 generations, with substitution and clock models unlinked between partitions. Convergence and ESS (>200) of parameters were assessed in Tracer. LogCombiner v. 10.5.0 was used to combine trees from the four runs, discarding the first 10% of generations as burn-in. A maximum clade credibility tree was processed in TreeAnnotator v. 10.5.0 with the posterior probability (PP) limit set to 0.5 and mean node heights summarized.

## Results

### Molecular results

**Figure 2.**
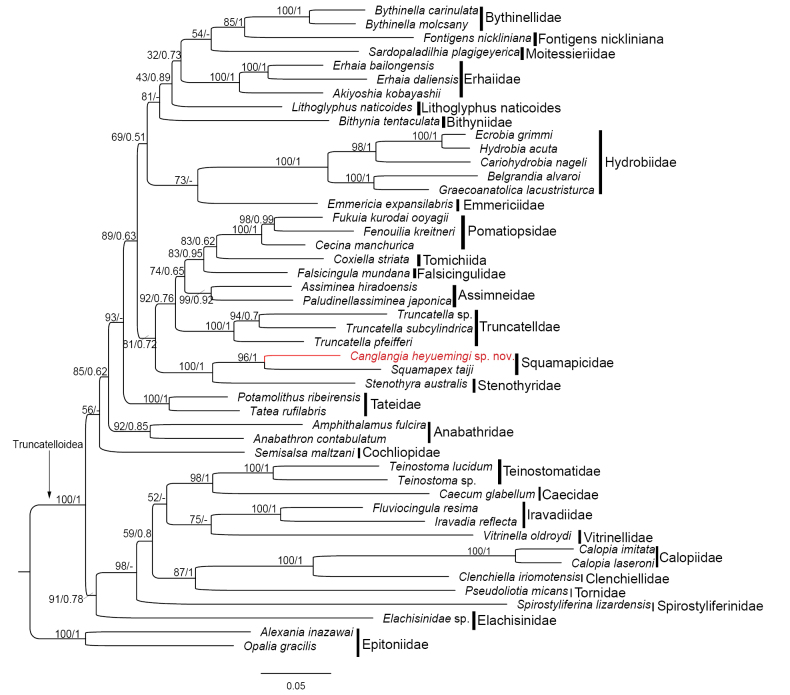
Maximum-likelihood tree of Truncatelloidea based on the combined dataset of four-gene sequences (COI, 16S, H3 and 28S). Numbers above branches indicate maximum-likelihood bootstrap support values and Bayesian posterior probabilities.

Molecular phylogenies inferred from both Bayesian inference (BI) and maximum likelihood (ML) analyses recovered the superfamily Truncatelloidea as a monophyletic group, subdivided into two major clades (BS = 100%, BPP = 1). Although relationships among some families within the superfamily received low support, the specimens collected from Luquan, Yunnan, China, together with *Squamapex taiji*, formed a strongly supported monophyletic clade (BS = 96%, BPP = 1) that is sister to *Stenothyra australis* (Fig. 2). Pairwise uncorrected COI p-distance analysis demonstrated genetic distance between this species and *Squamapex taiji* is 15%. These distances, as well as the distinct morphological characteristics, provide compelling evidence for its classification as a new genus and species *Canglangia heyuemingi* gen. et sp. nov. in the family Squamapicidae.

### Time-calibrated species tree

**Figure 3.**
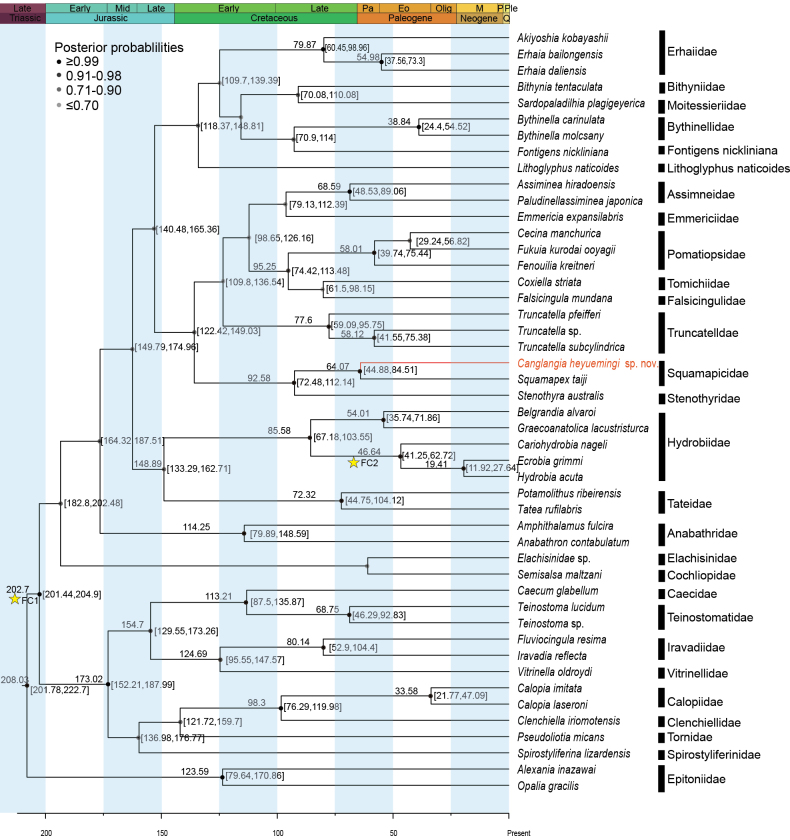
Maximum clade credibility tree based on four molecular markers (COI, 16S, H3 and 28S) obtained from BEAST. Calibration points are marked as FC1 and FC2. The new species is in red. Transversal stripes represent 25 million-year intervals. Numbers above the branches represent the mean age for the node directly below the branch (only marked when PP ≥ 0.91). Numbers between brackets represent the 95% confidence interval for the ages of the nodes directly above them. Overall posterior probabilities are shown on the nodes according to the legend. Species are attributed to families on the right.

The time-calibrated phylogenetic tree generated from the BEAST analysis was essentially congruent with the ML and BI phylogenetic trees. Molecular dating analyses reveal that the majority of family-level diversification within the superfamily Truncatelloidea occurred during the Cretaceous period. The split between the family Squamapicidae and Stenothyridae is estimated to have occurred approximately 92.58 Ma (95% HPD = 72.48–112.14 Ma). Within Squamapicidae, the stream-dwelling species *Canglangia heyuemingi* sp. nov. and the lake-endemic *Squamapex taiji* diverged later, around 64.07 Ma (95% HPD = 44.88–84.51 Ma) (Fig. 3).

### Systematics

**Family Squamapicidae L.-J. Zhang & von Rintelen, 2024**

#### Genus Canglangia

Taxon classification

Animalia

GastropodaSquamapicidae

H. Zheng, H.-Q. Xiang, L.-J. Zhang & X.-P. Wu gen. nov.

B2786561-3976-5467-AABD-04CD2F01DC1C

https://zoobank.org/43CF9792-85F1-46B4-8651-8656601EBC1D

##### Type species.

*Canglangia heyuemingi* H. Zheng, H.-Q. Xiang, L.-J. Zhang & X.-P. Wu, gen. et sp. nov.

##### Diagnosis.

Shell minute, solid; protoconch whorl upper side covered with many lines of regularly distributed large, square-scale-shaped protuberances, lower side with many regular spirals, teleoconch without complex microsculpture. Inner lip upper part with developed teeth; developed posterior canal. Operculum kidney-shaped, inner opercular region long crescent, with two different projections on the upper and lower parts of the inner opercular region.

##### Etymology.

The Latin name of this species is derived from the word ‘Canglang’ in the pre-Qin Chu region folk song Canglang Ge (Song of the Canglang), referring to the clear, flowing water in the species’ habitat. The term is feminine in gender. The Vernacular name is 沧浪螺 (cang lang luo).

##### Remarks.

The complex sculpture of the protoconch and the long crescent-shaped opercular region with two different projections on the upper and lower part of the inner opercular surface confirms the placement of the new taxon within Squamapicidae. This species is readily distinguished from *Squamapex taiji* by its developed posterior canal, long crescent-shaped inner opercular region, and distinct protoconch morphology.

#### Canglangia heyuemingi

Taxon classification

Animalia

GastropodaSquamapicidae

H. Zheng, H.-Q. Xiang, L.-J. Zhang & X.-P. Wu sp. nov.

9A0226A4-502B-5751-89B8-660CBA7D0061

https://zoobank.org/1286ECD9-0EE1-462A-9BFA-26D08542BEDA

Figs 4, 5, 6

##### Material examined. •

**Figure 4.**
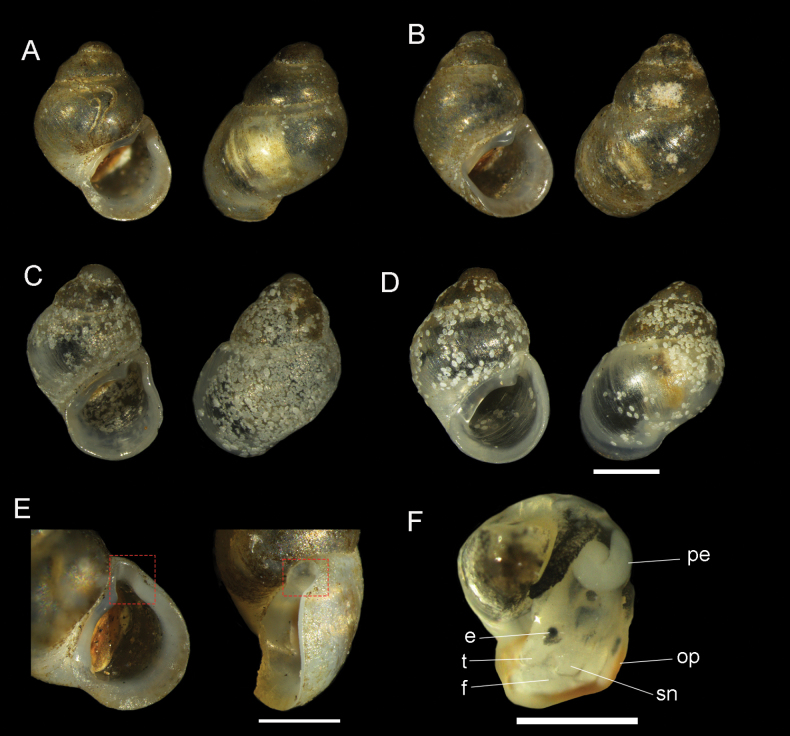
*Canglangia heyuemingi* gen. et sp. nov. **A**. Holotype, NCU_CLH251001; **B**–**D**. Paratypes, NCU_CLH251002–04; **F**. Head of male. Abbreviations: e, eye; t, tentacle; sn, snout; f, foot; op, operculum; pe, penis. Scale bars: 0.5 mm. The red box shows posterior respiratory canal.

***Holotype***: NCU_CLH251001, shell height 1.52 mm, shell width 1.07 mm (Fig. 4A), a spring near the Pudu River, Luquan Yi and Miao Autonomous County, Kunming City, Yunnan Province, China, 25.8925°N, 102.7411°E, in August 2025. • ***Paratypes***: 3 specimens, NCU_CLH251002–04, shell height 1.50–1.56 mm, shell width 1.07–1.14 mm (Fig. 4B–D), locality and habitat same as holotype.

**Figure 5.**
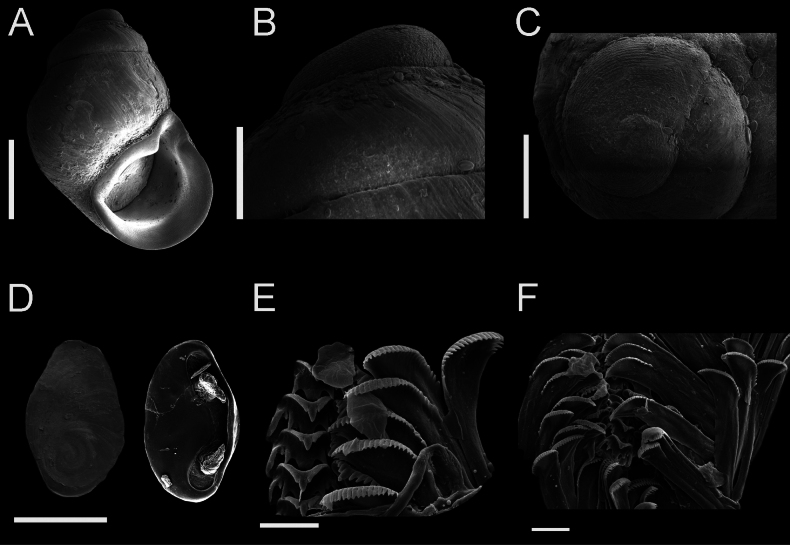
SEM photo of *Canglangia heyuemingi* gen. et sp. nov. **A**. SEM photo of the shell; **B, C**. SEM photo of the protoconch; **D**. SEM photo of the operculum; **E, F**. Photo of the radula. Scale bars: 0.5 mm (**A**); 0.2 mm (**B, C**); 0.3 mm (**D**); 5 µm (**E, F**).

**Figure 6.**
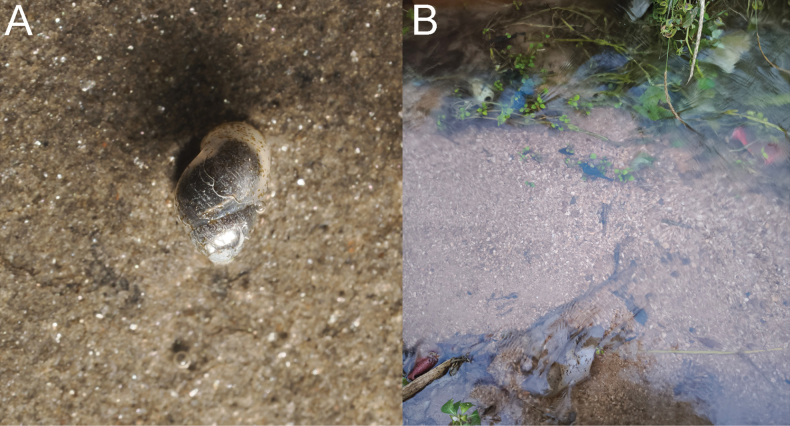
Live animals (**A**) and habitat (**B**) of *Canglangia heyuemingi* gen. et sp. nov.

##### Diagnosis.

Same as the genus.

##### Description.

Shell minute, dextral, oval, thin but solid, white and transparent; mostly with four to five whorls, protoconch whorl upper side covered with many lines of regularly distributed large, square-scale-shaped protuberances; teleoconch without complex microsculpture, with fine growth lines; body whorl large (Fig. 4A–D, 5A–C). Aperture oval; inner lip upper part with developed teeth; outer lip thin but solid; developed posterior canal (Figs 4E, 5A). Umbilicus close. Nacre not smooth (Figs 4A–D, 5A), with many short line bumps.

***Operculum*** (Fig. 5D) corneous, thin and fragile, kidney-shaped, transparent yellow in color; inner opercular region long crescent, with two different projections on the upper and lower part of the inner opercular region; upper projection little longer than lower projection, the lower end of upper projection and upper end of lower projection both pointing to the central of inner margin; enamel region flat and smooth, inner enamel region narrow and thickened; marginal region very thin and fragile.

***Radula*** (Fig. 5E, F) central tooth bearing one broad central denticle and one small sharp cusp on either side and with two to three small sharp cusps on central tooth base either side; lateral teeth with four to five cusps on the inner side and six cusps on the outer sides; inner marginal teeth with 23–25 cusps, outer marginal teeth with 19–21 cusps.

Snout slender, greyish white; tentacles slender, white. Soft body transparent pale grey. Mantle smooth, black; penis translucent white, coiled; foot small, anterior part expanded laterally and anterior edge indented (Fig. 4F).

##### Etymology.

The name of this species is in honor of Mr He Yueming, a conchologist who collected the specimens. The Vernacular name is 何氏沧浪螺 (he shi cang lang luo).

##### Habitat and distribution.

This species has been found only in springs near the aforementioned locality, where it lives on rocks and wood in shallow water (Fig. 6).

## Discussion

Highly ornamented protoconchs are exceptionally rare among strictly freshwater gastropods. Within the superfamily Truncatelloidea, only three species have been documented in China with this feature to date: *Squamapex taiji*, *Kunmingia tiejian* Chen, He, Xiang & Zhang, 2025, and *K. fireflyae* Lu, He, Chen & Zhang, 2025 (Zhang et al. 2024; Chen et al. 2025), all of which are endemic to Lake Fuxian. Field observations indicate that the habitats of *Kunmingia* and *Squamapex taiji* experience considerable wave activity, creating hydrodynamic conditions in certain areas of these ancient large lakes that functionally resemble small marine bays. The specialized shell structures of these species—analogous to those seen in marine gastropods—may therefore help reduce mechanical damage to their larvae under such dynamic conditions. Notably, *Kobeltocochlea martensiana* (Dybowski, 1875) and *Yaroslawiella eximia* Sitnikova, 2001 from Lake Baikal also display this trait (Sitnikova et al. 2021), suggesting that ornamented protoconchs may in some cases represent a neutral trait associated with ancient lake ecosystems. Squamapicidae is thought to have evolved from a common marine ancestor in the Tethys Ocean during the Late Cretaceous (Zhang et al. 2024). In marine environments, highly ornamented protoconchs may protect veliger larvae from mechanical stress (Hickman 1999), offering a plausible explanation for the retention of such shells in this family. Our phylogenetic and morphological analyses strongly support the establishment of a new genus within the previously monotypic Squamapicidae. The inclusion of the new genus in this family is well justified by the shared, complex protoconch sculpture—a synapomorphy of the family—indicating that this trait is more stable than previously recognized. However, as the only previously known species, *Squamapex taiji*, was restricted to Lake Fuxian, it remained unclear whether its protoconch structure resulted from adaptation to the ancient lake environment or was inherited from marine ancestors. By contrast, the newly discovered species is found exclusively in springs and not in nearby rivers, suggesting the absence of a veliger larval stage. Rearing observations further confirmed that neither *K. tiejian* nor *K. fireflyae* undergoes a veliger larval stage. Moreover, other *Kunmingia* species and related genera from Lake Fuxian show no evidence of highly ornamented protoconchs (Chen et al. 2025; He et al. 2025). From these lines of evidence, we infer that, at least in freshwater environments, highly ornamented protoconchs are not causally linked to the presence of veliger larvae.

The discovery of a well-developed posterior respiratory canal in the new genus represents a morphological innovation that challenges established paradigms in freshwater gastropod evolution. This innovation is underscored by our molecular dating analysis, which estimates the divergence between the stream-specific *Canglangia heyuemingi* gen. et sp. nov. and its lake-specific relative, *Squamapex taiji*, at approximately the Early Paleogene. This post-Cretaceous timing suggests that the evolutionary split and subsequent ecological specialization occurred shortly after the K-Pg boundary, a period marked by a mass extinction event that profoundly reshaped global ecosystems (Schulte et al. 2010). Such periods of environmental upheaval are known to create ecological opportunities, often triggering adaptive radiations in surviving lineages (Alroy 1999; Friedman and Sallan 2012). The divergence of *C. heyuemingi* and *S. taiji* may represent a localized example of this broader macroevolutionary pattern, where the extinction of incumbent species opened new niches within freshwater habitats, facilitating the ecological transition from stable lacustrine environments to dynamic mountain streams.

Field observations of the spring habitat of *C. heyuemingi* gen. et sp. nov. revealed a highly dynamic hydrological regime, which may explain the functional significance of this structure. During the drier spring season, when water discharge is minimal, individuals were frequently observed crawling out of the water onto moist rock surfaces. Under these conditions, the posterior canal may enhance capillary action at the shell aperture, thereby aiding in water retention and preventing desiccation during aerial exposure. Conversely, in autumn, when water flow intensifies, these snails remain firmly attached to rocks in stronger currents. In this context, the canal likely functions as an effective excurrent siphon, facilitating waste expulsion and maintaining respiratory efficiency under high-flow conditions. This structural feature opens new avenues for investigating the functional morphology and ecological adaptations of freshwater snails to specialized and fluctuating niches. The ecological divergence is further emphasized by the distinct shell morphology and unique possession of the respiratory canal, which likely enabled the new genus to exploit a different ecological zone within the freshwater ecosystem, potentially reducing competition with its relatives. The ecological transition from the stable environment of an ancient lake to the dynamic conditions of mountain streams underscores a major evolutionary radiation within Squamapicidae, reflecting their remarkable adaptive capacity.

Recent years have witnessed the discovery of numerous new Truncatelloidean species across Yunnan (Chen et al. 2025; Xiang et al. 2025), indicating that a substantial diversity of microsnails remains to be documented. However, due to their limited dispersal capacity and high habitat specificity—traits common among freshwater microgastropods (Ponder and Colgan 2002; Strong et al. 2008), many of these species were already facing severe threats of extinction at the time of their discovery (Neubauer et al. 2021; He et al. 2025). Our findings further underscore the rich diversity of freshwater gastropods in this region and highlight the critical need for continued exploration of its ancient lakes and spring systems. Such research is essential to fully understand and effectively conserve these unique and vulnerable Truncatelloidean lineages.

## Supplementary Material

XML Treatment for Canglangia

XML Treatment for Canglangia heyuemingi

## Additional information

### Artificial Intelligence (AI) use

The authors accept full responsibility for the content of the manuscript, including the disclosure of any use of AI.

No AI tools were used in the preparation of this manuscript.

### Author ORCIDs

H. Zheng https://orcid.org/0009-0004-5222-3975

L.-J. Zhang https://orcid.org/0000-0002-6002-873X

Z.-G. Chen https://orcid.org/0000-0003-2689-3321

H.-Q. Xiang hhttps://orcid.org/0009-0005-6737-3092

X.-C. Huang https://orcid.org/0000-0002-0242-3571

X.-P. Wu https://orcid.org/0000-0002-8037-5640

### Data availability

The data that support this study are available in the article and accompanying online Supplementary material.

## Supplementary materials

Supplementary material 1 GenBank accession numbers used in this study Data type xlsx Explanation note Sequences obtained for this study are marked by an asteris. https://binary.pensoft.net/file/1675863This dataset is made available under the Open Database License (http://opendatacommons.org/licenses/odbl/1.0/). The Open Database License (ODbL) is a license agreement intended to allow users to freely share, modify, and use this Dataset while maintaining this same freedom for others, provided that the original source and author(s) are credited. Hui Zheng, Yu-Hui Xing, Le-Jia Zhang, Qian Li, Zhong-Guang Chen, Hong-Quan Xiang, Shan Ouyang, Xiao-Chen Huang, Xiao-Ping Wu

## References

[B1] Alroy J (1999) The fossil record of North American mammals: evidence for a Paleocene evolutionary radiation. Systematic Biology 48(1): 107–118. 10.1080/106351599260472 12078635

[B2] Castresana J (2000) Selection of conserved blocks from multiple alignments for their use in phylogenetic analysis. Molecular Biology and Evolution 17: 540–52. 10.1093/oxfordjournals.molbev.a026334 10742046

[B3] Chen H, Shi BY, Du LN, Sun HY (2023) Description of a new species of *Hua* (Gastropoda: Semisulcospiridae) from Guizhou, China, based on morphology and molecular evidence. Zoological Science 40(5): 414–-421. 10.2108/zs230025 37818890

[B4] Chen H, Zhang LJ, He YM, Xiang HQ, Lu YZ, Li CY, Yao YT, Gao H, Huang XC, Wu XP (2025) Diversity in an ancient lake: taxonomic and phylogenetic insights from eight new freshwater snail species (Triculinae: Pomatiopsidae) of Lake Fuxian, Southwest China. Zoological Journal of the Linnean Society 205(2): zlaf130. 10.1093/zoolinnean/zlaf130

[B5] Colgan DJ, Ponder WF, Eggler PE (2000) Gastropod evolutionary rates and phylogenetic relationships assessed using partial 28S rDNA and histone H3 sequences. Zoologica Scripta 29: 29–63. 10.1046/j.1463-6409.2000.00021.x

[B6] Colgan DJ, Ponder WF, Beacham E, Macaranas JM (2003) Gastropod phylogeny based on six segments from four genes representing coding or non-coding and mitochondrial or nuclear DNA. Molluscan Research 23: 123–48. 10.1071/MR03002

[B7] Cummings KS, Lydeard C (2019) Freshwater Mollusks of the World: a Distribution Atlas. Baltimore: Johns Hopkins University Press, 242 pp. 10.1353/book.66164

[B8] Czaja A, Cardoza-Martnez GF, Becerra-Lpez JL, Estrada-Rodrguez JL, Alonzo-Rojo F, Vila-Rodrguez V, Valenzuela-Garca AA (2025) Worlds smallest freshwater snail? A new genus and species of subterranean snail (Gastropoda, Cochliopidae) with extremely tiny shell from Los Chorros, Coahuila, northern Mexico. Zootaxa 5660(3): 413–425. 10.11646/zootaxa.5660.3.7 41119866

[B9] Dillon RT (2000) The Ecology of Freshwater Molluscs. Cambridge, UK: Cambridge University Press, 524pp. 10.1017/cbo9780511542008

[B10] Du LN, Köhler F, Yu GH, Chen XY, Yang JX (2019) Comparative morpho-anatomy and mitochondrial phylogeny of Semisulcospiridae in Yunnan, south-western China, with description of four new species (Gastropoda: Cerithioidea). Invertebrate Systematics 33(6): 825–848. 10.1071/IS18084

[B11] Edgar RC (2004) MUSCLE: multiple sequence alignment with high accuracy and high throughput. Nucleic Acids Research 32: 1792–7. 10.1093/nar/gkh340 PMC390337 15034147

[B12] Folmer O, Black M, Hoeh W, Lutz R, Vrijenhoek R (1994) DNA primers for amplification of mitochondrial cytochrome c oxidase subunit I from diverse metazoan invertebrates. Molecular Biotechnology 3: 294–299. 7881515

[B13] Friedman M, Sallan LC (2012) Five hundred million years of extinction and recovery: a Phanerozoic survey of large‐scale diversity patterns in fishes. Palaeontology 55(4): 707–742. 10.1111/j.1475-4983.2012.01165.x

[B14] He YM, Xiang HQ, Gao H, Lv AS, Chen H (2025) Two new genera and five new species of freshwater snails (Gastropoda: Truncatelloidea: Pomatiopsidae) from the Plateau ancient Lake, Yunnan Province, China. Animal Taxonomy and Ecology 71: 345–57. Advance online publication. 10.1556/1777.2025.00066

[B15] Hickman CS (1999) Adaptive function of gastropod larval shell features. Invertebrate Biology 118: 346–56. 10.2307/3227006

[B16] Hillis DM, Dixon MT (1991) Ribosomal DNA: molecular evolution and phylogenetic inference. The Quarterly Review of Biology 66: 411–53. 10.1086/417338 1784710

[B17] Jirapatrasilp P, Sutcharit C, Panha S (2022) Annotated checklist of the operculated land snails from Thailand (Mollusca, Gastropoda, Caenogastropoda): the family Pupinidae, with descriptions of several new species and subspecies, and notes on classification of Pupina Vignard, 1829 and Pupinella Gray, 1850 from mainland Southeast Asia. ZooKeys 1119: 1–115. 10.3897/zookeys.1119.85400 PMC9848625 36762355

[B18] Jirapatrasilp P, Tongkerd P, Páll-Gergely B, Lee CT, Panha S, Becher E, Hausdorf B, Sutcharit C (2025) Molecular phylogeny of the operculated land snail family Pupinidae (Caenogastropoda, Cyclophoroidea) in mainland Southeast Asia. Zoologica Scripta 54(4): 526–547. 10.1111/zsc.12727

[B19] Katoh K, Toh H (2008) Recent developments in the MAFFT multiple sequence alignment program. Briefings in Bioinformatics 9: 286–298. 10.1093/bib/bbn013 18372315

[B20] Kessing B, Croom H, Martin A, Mcintosh C, Owen Mcmillan W, Palumbi S (1989) The simple fools guide to PCR. University of Hawaii, Honolulu, 45 pp.

[B21] Kumar S, Stecher G, Li M, Knyaz C, Tamura K (2018) MEGA X: molecular evolutionary genetics analysis across computing platforms. Molecular Biology and Evolution 35: 1547–1549. 10.1093/molbev/msy096 PMC5967553 29722887

[B22] Lanfear R, Frandsen PB, Wright AM, Senfeld T, Calcott B (2017) Partitionfinder 2: new methods for selecting partitioned models of evolution for molecular and morphological phylogenetic analyses. Molecular Biology and Evolution 34: 772–773. 10.1093/molbev/msw260 28013191

[B23] Latiolais JM, Taylor MS, Roy K, Hellberg ME (2006) A molecular phylogenetic analysis of strombid gastropod morphological diversity. Molecular Phylogenetics and Evolution 41: 436–444. 10.1016/j.ympev.2006.05.027 16839783

[B24] Minh BQ, Schmidt HA, Chernomor O, Schrempf D, Woodhams MD, von Haeseler A, Lanfear R (2020) IQ-TREE 2: New models and efficient methods for phylogenetic inference in the genomic era. Molecular Biology and Evolution 37(5): 1530–1534. 10.1093/molbev/msaa015 PMC7182206 32011700

[B25] Neubauer TA, Hauffe T, Silvestro D, Schauer J, Kadolsky D, Wesselingh FP, Harzhauser M, Wilke T (2021) Current extinction rate in European freshwater gastropods greatly exceeds that of the late Cretaceous mass extinction. Communications Earth & Environment 2(1): 97. 10.1038/s43247-021-00167-x

[B26] Nützel A (2014) Larval ecology and morphology in fossil gastropods. Palaeontology 57: 479–503. 10.1111/pala.12104

[B27] Ponder WF, Colgan DJ (2002) What makes a narrow-range taxon? Insights from Australian freshwater snails. Invertebrate Systematics 16(4): 571–582. 10.1071/it01043

[B28] Ronquist F, Teslenko M, van der Mark P, Ayres DL, Darling A, Höhna S, Larget B, Liu L, Suchard MA, Huelsenbeck JP (2012) MrBayes 3.2: Efficient Bayesian phylogenetic inference and model choice across a large model space. Systematic Biology 61(3): 539–542. 10.1093/sysbio/sys029 PMC3329765 22357727

[B29] Schulte P, Alegret L, Arenillas I, Arz JA, Barton PJ, Bown PR, Bralower TJ, Christeson GL, Claeys P, Cockell CS, Collins GS, Deutsch A, Goldin TJ, Goto K, Grajales-Nishimura JM, Grieve RAF, Gulick SPS, Johnson KR, Kiessling W, Koeberl C, Kring DA, MacLeod KG, Matsui T, Melosh J, Montanari A, Morgan JV, Neal CR, Nichols DJ, Norris RD, Pierazzo E, Ravizza G, Rebolledo-Vieyra M, Reimold WU, Robin E, Salge T, Speijer RP, Sweet AR, Urrutia-Fucugauchi J, Vajda V, Whalen MT, Willumsen PS (2010) The Chicxulub Asteroid Impact and Mass Extinction at the Cretaceous-Paleogene Boundary. Science 327(5970): 1214–1218. 10.1126/science.1177265 20203042

[B30] Sitnikova T, Teterina V, Maximova N, Kirilchik S (2021) Discordance of genetic diversification between deep‐and shallow‐water species of Kobeltocochlea Lindholm, 1909 (Caenogastropoda: Truncatelloidea: Benedictiidae) endemic to Lake Baikal with the description of a new species, review of the genus, and notes on its origin. Journal of Zoological Systematics and Evolutionary Research 59(8): 1775–1797. 10.1111/jzs.12545

[B31] Strong EE, Gargominy O, Ponder WF, Bouchet P (2008) Global diversity of gastropods (Gastropoda; Mollusca) in freshwater. Hydrobiologia 595: 149–166. 10.1007/s10750-007-9012-6

[B32] Xiang HQ, Lu YZ, He YM, Li CY, Gao H, Lv AS, Chen H (2025) Twenty-seven new species and four new genera of Gastropoda (Animalia: Mollusca) from plateau lake Quaternary sediments in Yunnan, southwestern China. Ecologica Montenegrina 84: 48–71. 10.37828/em.2025.84.6

[B33] Xiang HQ, Chen LRX, Wang BY, He YM, Gao H, Chen H, Lv AS, Zha XC, Xie TX, Zheng J, Yang SZ, Wang P (2026) Fifteen new species of freshwater snail fossil Viviparidae (Gastropoda: Architaenioglossa) from the Neogene and Quaternary of the Yunnan Province, China. Animal Taxonomy and Ecology 72(1): 76–109. 10.1556/1777.2026.00126

[B34] Zhang D, Gao F, Jakovlić I, Zou H, Zhang J, Li WX, Wang GT (2020) PhyloSuite: an integrated and scalable desktop platform for streamlined molecular sequence data management and evolutionary phylogenetics studies. Molecular Ecology Resources 20: 348–55. 10.1111/1755-0998.13096 31599058

[B35] Zhang LJ, Bernardes SC, Meng K, von Rintelen T (2024) A new family of freshwater snails with Cretaceous origin from Yunnan, China. Zoological Journal of the Linnean Society 202, zlae117. 10.1093/zoolinnean/zlae117

[B36] Zheng H, Zhang LJ, Zhang YY, Xiang HQ, He YM, Ouyang S, Wu XP (2026) Multilocus phylogeny reveals new species from the ancient Lake Fuxian and synonymy of *Cipangopaludina* with *Margarya* (Gastropoda: Viviparidae). Zoologica Scripta. 10.1111/zsc.70050

